# Planetary health and emancipatory worlding

**DOI:** 10.1177/17579759251378432

**Published:** 2025-10-24

**Authors:** Massih Zekavat

**Affiliations:** University of Groningen, the Netherlands

**Keywords:** anthropocentrism, multispecies planetary health, capitalism

## Abstract

This study critiques the prevailing anthropocentric and neoliberal frameworks within planetary health discourses, advocating for multi-species wellbeing – a departure from the dominant models of planetary health that tend to prioritize human interests and overlook the symbiotic relationships among holobionts. Drawing on posthumanism and relational theories to critique anthropocentrism and promote emancipatory worlding, it highlights the role of socio-economic disparities in environmental degradation and the unequal distribution of power and responsibility. This study further reveals the inadequacy of resilience and adaptation strategies often promoted by neoliberal agendas, calling instead for systemic change. True planetary health requires a shift from anthropocentric paradigms to a symbiotic model that incorporates material justice and restitution. Addressing global health disparities and environmental degradation must move beyond market-based solutions, prioritizing the interconnectedness of all species and their environments.

## Introduction

This paper calls for a more thorough and radical rethinking of the ongoing discourse surrounding planetary health. It reexamines presuppositions in its dominant conceptualizations, which often remain limited by anthropocentric and Western neoliberal frameworks, and instead advocates a more equitable and holistic approach that recognizes relationality and symbiotic interdependence between human and more-than-human.

I further explore possibilities for a non-anthropocentric, though still anthropomorphic, approach to health, underscore the common but differentiated responsibilities of various communities and socio-economic classes, and critique the unequal distribution of accountability and power, particularly how inequalities in decision-making and access to resources create disparities in addressing ecological and environmental urgencies. Going beyond current anthropocentric and market-based solutions, this research advocates emancipatory worlding, restitution, and multispecies wellbeing.

## Theory and methods

By challenging anthropocentric and speciesist paradigms, this research seeks to envision a flattened ontology that goes beyond human-centered perspectives. The methodological approach is informed by two key theoretical paradigms: posthumanism and the relational turn in sustainability studies.

Posthumanism serves as a guiding framework to critique anthropocentrism, decenter human subject and experience, and embrace the interconnectedness of humans and more-than-humans. This involves a critical rethinking of agency, identity and subjectivity, employing interdisciplinary and non-linear approaches to explore more-than-human agencies and interrelations. Posthumanism is particularly pertinent in decentring *Homo sapiens* for the purpose of this study (1–4).

The relational turn in sustainability science emphasizes a paradigm shift from viewing the world as composed of discrete entities to understanding it as co-constituted by dynamic interdependant relationships across material, ecological, social, cultural, political and economic dimensions. This approach rejects mechanistic and dualistic views, advocating for acknowledging and cherishing interconnectedness, embodied experiences and inclusion of indigenous perspectives, while emphasizing decolonial thinking alongside symbolic and affective power (5–10).

The study examines the conceptualizations of planetary health in scholarly literature and key documents to illustrate the relational turn and posthumanist thinking in practice. The documents were selected based on their prominence in the discourse of planetary health and their cultural diversity. ‘Safeguarding human health in the Anthropocene epoch’ helps interrogate anthropocentric biases and explore how more-than-human agencies are marginalized (11). ‘People’s Agreement of Cochabamba’ exemplifies an alternative paradigm informed by relational thinking (12). English language is a major limitation of this study.

### Situatedness of halobionts and their symbiotic interdependence

Many approaches to planetary health seem to hesitate in fully embracing the shifting paradigm in that they remain to be anthropocentric and shy away from radically departing from prioritizing (neoliberal) economic interests.

### Anthropocentrism

Anthropocentrism may pose a significant barrier to the widespread adoption of multispecies planetary health. Defined as a system of beliefs and practices that position human beings as the central or most significant entities in the universe, anthropocentrism prioritizes human needs, interests and values over those of other living beings, material actants and the environment.

Anthropocentrism frequently prioritizes instrumental value, assessing the worth of entities primarily through their utility to humans, particularly in economic terms, which can foster extractivist practices. By contrast, the concept of intrinsic value asserts that entities possess inherent worth, irrespective of their utility to humans. This perspective advocates and cherishes more-than-human existence based on its inherent value, thereby aligning with sustainable practices that prioritize planetary health.

A *Lancet* editorial, for instance, that aims to provide ‘The bigger picture of planetary health,’ defines planetary health as ‘the health of human civilization and the state of the natural systems on which it depends’ (13). Putting *Homo sapiens* and their health at the center inevitably reiterates anthropocentric attitudes at the cost of the symbiotic interrelationships among all holobionts and the web of socio-political dynamics that have impacted them.

Many more attempts at conceptualizing and defining planetary health seem to be susceptible to the same drawback (14–17). Martens strives to do better justice to the concept by decentering *Homo sapiens* and underscoring symbiotic interrelations in writing, ‘Planetary health can . . . be defined as a holistic systems view of the world, recognizing the interconnectedness of the health and wellbeing of all life on Earth and the need to regenerate natural systems, rather than simply minimizing harm’ (18). Nevertheless, a careful reading will soon reveal that mastery is secured for human beings. Instead of ascribing subjectivity to Earth and natural systems by unhesitatingly putting them in the subject position, the definition relegates them to the position of objects that need to be regenerated, presumably by human intervention.

‘Safeguarding human health in the Anthropocene epoch’ is one of the most frequently cited documents that defines planetary health as ‘the achievement of the highest attainable standard of health, wellbeing and equity worldwide through judicious attention to the human systems – political, economic and social – that shape the future of humanity and the Earth’s natural systems that define the safe environmental limits within which humanity can flourish’ (11). It concentrates on human systems, their future, their safe environment and their flourishing. The definition remains silent about the role of other holobionts and their symbiotic relationships with *Homo sapiens*. The writers continue, ‘Put simply, planetary health is the health of human civilization and the state of the natural systems on which it depends.’ The state of natural systems, in other words, do not have any intrinsic value; they fall into the circumference of this definition as long as their use value serves the health of human civilization.

Biocentrism and ecocentrism offer promising alternatives to anthropocentrism for expanding our understanding of planetary health and recognizing the profound interdependence of human and more-than-human wellbeing. These perspectives depart from instrumental rationality, a philosophical concept that refers to the use of appropriate means to achieve specific ends. Instrumental rationality is critiqued for its role in reducing diverse forms of value – cultural, ethical and ecological – to economic utility under capitalist systems (19,20). By moving beyond this narrow framework, biocentrism and ecocentrism encourage a more holistic understanding of the world, where the intrinsic value of ecosystems and species is acknowledged and prioritized.

Moreover, a departure from anthropocentrism acknowledges that human beings are holobionts – complex entities composed of multiple species that coexist and interact symbiotically. A holobiont is not a single organism but rather a collective, encompassing the host, its microbiome and the surrounding environment. This understanding emphasizes the profound interdependence of human life with other species and ecosystems, challenging the notion of humans as autonomous, self-contained subjects.

Likewise, Morton’s concept of mesh describes the intricate web of natureculture interdependence. Morton argues that ‘nothing exists all by itself, and so nothing is fully “itself”’ (21). Mesh dissolves the boundaries between human and more-than-human, culture and nature, revealing that all entities are enmeshed in relationships of mutual confluence and interdependence. By embracing this view, we move beyond anthropocentrism and recognize the deeply interconnected nature of life as central to multispecies planetary health.

Such paradigm shift and its implications for redistributing agency may raise concerns about the accountability of human beings in addressing the anthropogenic crisis. It might seem that humans must be placed at the center to be held accountable, as no other entity within this comprehensive system can act on their behalf. However, acknowledging material agency does not exclude human responsibility. The tension between individual and material agency does not contradict anthropogenic climate change and environmental degradation; rather, it underscores the necessity for behavioral change. This holistic perspective encourages individuals to recognize their roles as holobionts, interconnected with other species and material agents, which fosters a deeper understanding of kinships, symbiotic relationships and interdependencies. It is not about deflecting responsibility but about recognizing that agency extends beyond the individual. We often fail to fully appreciate the agency, autonomy and sentience of the more-than-human in relation to human existence, actions and institutions. The challenge lies in conveying the dynamic and responsive nature of the vibrant matter that constitutes the critical zones we inhabit as holobionts.

While *Homo sapiens* must assume responsibility and act urgently, they are not the sole agents in this process. Emphasizing individual responsibility, however, presents two critical limitations. First, it risks obscuring the role of heteronomy and the systemic transformations required for meaningful progress. It inadequately acknowledges the unequal contributions of different communities to climate change, as well as the disparities in their capacity to respond.

Second, it overlooks material agency. Ghosh critiques this oversight by exploring the agency of more-than-human actors, such as nutmegs (22), tea, and opium (23), as historical forces that shape events independently of human intentions. He underscores the challenges of conceptualizing human-plant interactions, which are profoundly shaped by class and power differentials under imperial rule. He further highlights recent climate-intensified events as evidence of the vital, agentive properties of more-than-human forces (biological, geological, and atmospheric). Though these forces may appear temporarily subdued by human control, they retain the capacity to assert their autonomy and dominance beyond critical thresholds.

Thus, it is essential to move away from anthropocentrism. By acknowledging material agency and the interspecies symbiotic interdependence that defines us as holobionts, we can foster a more holistic understanding of our place on the planet – one that encourages a shift in how we perceive and interact with our environment, other species and vibrant matter, emphasizing the importance of nurturing and respecting the complex web of relationships that sustain life on Earth.

### Neo-liberal capitalism

The Rockefeller definition does not fail only in acknowledging and treasuring symbiotic interrelations due to its anthropocentric approach, but also remains disturbingly silent about common but differentiated responsibility and restitution. It tends to generalize a certain experience – that of white, middle-aged, middle-class Western men – to humanity and human civilizations. This homogenizing attitude is partly driven by the economic imperatives inherent in planetary health. This is also reflected in the use of the term ‘Anthropocene era’ regardless of the pertinent critique of the term (24). Among others, Ghosh critiques the Anthropocene for its oversimplification of human accountability regarding environmental issues, arguing it unjustly implicates all humanity while ignoring significant historical inequalities in greenhouse gas emissions. He posits that the problems attributed to the Anthropocene stem primarily from colonialism, imperialism and capitalism, rather than human omnipotence (22,25). Consequently, many alternative framings have been proposed such as Capitalocene, (26) Chthulucene and Plantationocene (27).

In the same vein, Clark calls for ‘Another governance priority . . . to encourage the private sector to protect the environment and human health as part of doing business’ (28). This perspective, rooted in neoliberal ideology, places undue faith in free-market solutions while neglecting issues of equity. Neoliberalism refers to ‘a politico-economic doctrine that embraces robust liberal capitalism, constitutional democracy and a modest welfare state’ (29). By prioritizing private sector involvement without addressing inherent inequalities in resource distribution and access, we risk perpetuating existing inequalities rather than fostering meaningful change for both environmental and public health outcomes.

Similar well-intentioned but arguably misguided attempts emerge when planetary health advocates uncritically promote resilience (30). Promoting resilience within the planetary health discourse reveals a fundamental compliance to the status quo, often promoting a passive response to systemic crises. As noted by Chaudhary (31), resilience strategies – characterized by traits such as optimism, creativity and a cohesive life narrative – serve to reinforce existing ideologies that normalize suffering and prepare individuals for ongoing degradation. This ideological prescription is particularly troubling, as it can be seen as a means to sustain business-as-usual practices, effectively delaying necessary structural changes. In contrast, a progressive climate realism advocates for externalization and politicization, recognizing the collective political potential of those who are exhausted by the inequities of capitalism. The Exhausted, marked by a shared experience of degradation, can form a genuine political consciousness through recognition of their plight and resistance against oppressive systems. Rather than accepting resilience as a solution, this perspective emphasizes the importance of rebellion and the urgent need for transformative action in the face of ecological and socio-political crises.

Anthropocentrism and resilience remain to be the drawbacks of the much more comprehensive and progressive ‘From public to planetary health: a manifesto’ (32). The Manifesto posits,

Planetary health is an attitude towards life and a philosophy for living. It emphasizes people, not diseases, and equity, not the creation of unjust societies. We seek to minimize differences in health according to wealth, education, gender, and place. We support knowledge as one source of social transformation, and the right to realize, progressively, the highest attainable levels of health and wellbeing.

It continues to unequivocally link health with consumer capitalism in asserting, ‘Our patterns of overconsumption are unsustainable and will ultimately cause the collapse of our civilization. . . . We have created an unjust global economic system that favors a small, wealthy elite over the many who have so little.’

The Manifesto strives to eschew universalizing attitudes and underlines the role of economic, political and social institutions. Instead of advocating for market-based solutions, it criticizes the logic behind them. It clarifies that, ‘Our tolerance of neoliberalism and transnational forces dedicated to ends far removed from the needs of the vast majority of people, and especially the most deprived and vulnerable, is only deepening the crisis we face.’ This leads the authors to observe that we inhabit a world in which trust among individuals, institutions and leaders is deteriorating to a degree that undermines the foundations of peaceful and equitable societies, thereby fostering widespread disillusionment with democracy and the political system.

The Manifesto, therefore, calls for nothing less than a thorough rethinking of our relationship with each other and with institutions. It insists on intersectionality and collective action, and envisions new forms of cooperation. In the words of the authors, it calls for nothing less than ‘a new principle of planetism and wellbeing.’

While the Manifesto has received its fair share of criticism (33), most reactions seem to applaud and endorse its progressive visions. *Lancet* revisits the Manifesto after eight years (34), and Burkle calls for a global treaty based on the Manifesto (35). Schuftan et al. double down on its promises and express their dismay that ‘sustainable development initiatives designed especially for Asia and Africa still almost invariably involve the private sector, as lead partners of UN agencies and governments. But, in reality, the private sector comprises the very transnational corporations whose actions are in conflict with public health’ (36).

What seems to be missing in the discussion is a more rigorous attempt to eschew anthropocentrism, picturing *Homo sapiens* as enmeshed with all other beings in the critical zone, as well as a critique of promoting resilience. Moreover, the Manifesto yearns for a new world order, but shies away from suggesting an alternative. Beaglehole, one of the co-authors of the Manifesto, resounds the call for sustainable human development, but falls short of referring to Māori concept of Waiora, although he is based in New Zealand (37).

### People’s Agreement of Cochabamba

The ‘People’s Agreement of Cochabamba’ promotes a similarly progressive and equitable approach (12). It renounces anthropocentrism, colonialism, imperialism and socio-economic inequality in conceptualizing wellbeing across different species. It critiques the capitalist system as a primary cause of climate change, arguing that it promotes rabid overconsumption, unbridled exploitation of natural resources, and commodification of life. The Agreement calls for a systemic shift towards a sustainable, equitable model that harmonizes human existence with nature.

The Agreement identifies (racial) capitalism, colonialism and imperialism as major causes of environmental degradation and calls for an alternative system that fosters harmony with nature. It holds developed nations accountable for their historical contribution to greenhouse gas emissions and urges them to compensate developing countries through the reduction of emissions, technology transfer and restoration of ecosystems. The call for reparation of climate debt is coupled with support for indigenous and ancestral knowledge in achieving climate justice and sustainable development, especially regarding agriculture and food sovereignty. It advocates a Universal Declaration of the Rights of Mother Earth, recognizing the Earth as a living entity, endowed with inherent rights to life, respect, clean air, water, and freedom from pollution and genetic modification. T﻿he Agreement calls for the establishment of a global grassroots movement to champion these rights, mobilize public support, and compel governments to adopt environmentally just policies.

In binding wellbeing with justice and sociopolitical reparations, the Agreement denounces market-based solutions, as they tend to commodify nature and fail to address the root causes of climate change. The principles endorsed by the Agreement call on the field of planetary health to move beyond its reliance on neoliberal mechanisms, instead advocating for community-driven, non-commodified solutions to environmental and health crises. Another key implication for planetary health is the expansion of its focus: rather than prioritizing solely scientific and technological solutions, the Agreement emphasizes the critical role of indigenous knowledges in sustaining multispecies wellbeing.

The Agreement takes a much more critical and cautious approach towards adaptation and resilience. In part, it reads,

We the peoples have the equal right to be protected from the adverse effects of climate change and reject the notion of adaptation to climate change as understood as a resignation to impacts provoked by the historical emissions of developed countries, which themselves must adapt their modes of life and consumption in the face of this global emergency. We see it as imperative to confront the adverse effects of climate change, and consider adaptation to be a process rather than an imposition, as well as a tool that can serve to help offset those effects, demonstrating that it is possible to achieve harmony with nature under a different model for living.

The Agreement, thus, pushes for a more radical and fundamental transformation by framing environmental and health issues within broader systemic and ideological changes. In doing so, it challenges the more moderate, reformist approaches of planetary health and suggests that achieving true planetary health requires not just dethroning humans but also economic justice, systemic reform and an ethical commitment to nature as a living entity deserving of rights.

## Discussion and conclusion

I contended that imperialism, colonialism, capitalism and the predominance of a transactional rather than relational world view as major roots of our current existential crisis have not received ample attention in the conceptual frameworks of planetary health.

One potential counterargument could be that the analysis does not account for human history prior to European and American expansion, particularly whether there existed non-capitalist but equally rapacious civilizations or alternative constructs of sustainable and harmonious ecosystems. However, the narrative structure of declensionism – or the idea of a decline from a preferable or ideal state – risks implying that there once existed a harmonious relationship between humans and nature, which we might nostalgically attempt to reclaim. Such a perspective risks oversimplifying the past and distracting from the unprecedented nature of the contemporary crisis. While historical studies of civilizations that collapsed due to ecological overexploitation are instructive (38–45), the current crisis is distinct in its global scope, scale and unrivalled existential stakes, threatening not just individual civilizations but all life on Earth. It goes beyond how pre-capitalist societies depleted their resources into how practices such as expansionism, imperialism, expropriation, extractivism and environmental transformation – while present in earlier societies – have been intensified and globalized exponentially under capitalism. Thus, while anthropogenic ecological disruption predates capitalism, its scale and severity have reached unprecedented levels under the capitalist system.

While some might imply that individual consciousness and responsibility are sufficient to address the ecological and socio-political issues stemming from capitalism, this perspective represents a misguided shift from institutional accountability to individual action. As Saito emphasizes (46), changes in consumer consciousness are often co-opted by capital due to our deep entanglement in an economy that perpetually seeks growth. Marx referred to this phenomenon as ‘subsumption’ – a process through which capital absorbs societal changes, enveloping our lifestyles and leaving individuals powerless to effect systemic change.

This relentless entanglement fosters a socio-economic reality in which even waste and destruction are commodified as business opportunities – consider the rise of carbon offsetting and credits. As long as these mechanisms contribute to the creation of scarcity, they are seen as viable tools for generating profit. Saito aptly notes, ‘Climate change is, in this sense, a business opportunity like no other. It renders water, farmland, and habitable land scarce. As scarcity rises, so does demand, until it surpasses supply, creating a prime opportunity for capitalists to reap enormous profits.’ This dynamic underscores how capitalism not only exacerbates environmental crises but also thrives on them, transforming even the most pressing threats into avenues for accumulation.

Even more concerning is the way capital employs artificial scarcity through monetary scarcity caused by debt to solidify its domination. The consumption process under capitalism is driven by an endless stimulation of needs, which leads not to wealth but to the accumulation of loans. This burden of debt locks workers into a cycle of obedience, effectively making them pawns of the capitalist system. Here, the entanglement of environmental and socio-economic factors becomes evident as financial capitalism and debt are used to reinforce class structures within society.

The notion of multispecies planetary health must acknowledge that wealthier, more industrialized nations and communities are historically responsible for the majority of environmental damage, yet it is often marginalized communities that bear the brunt of the consequences. Natural systems are inevitably impacted by society, economy, politics and culture, and at the same time constitute them in their turn. In other words, human health, biological health, planetary health and social, political, economic and cultural dimensions of our existence are intertwined and co-constitutive. At the same time, multispecies planetary health must remain vigilant and critical of anthropocentrism and market-based solutions that risk exacerbating, rather than addressing, environmental issues in the long run.

Addressing the challenges facing human and more-than-human existence requires more than simply advocating for new perspectives and ethics. Current economic, legal and political systems appear to be at odds with the wellbeing of both human and ecological communities. These systems are sustained by entrenched political and economic interests, creating incentive structures that hinder even powerful actors from initiating change. Therefore, a genuine approach to planetary health must aim for nothing less than emancipatory worlding (24,47). This involves, among others, valuing both biological diversity and diversity within social institutions to counteract capitalism’s homogenizing tendencies.

A bold reimagining of planetary health further suggests embracing the notion of collapse at ‘the end of times’ (48). Instead of viewing collapse as something to be prevented, it can be perceived as both an inevitable process that is already unfolding and a necessary development, in the wake of undefeated despair at existing institutions and structures that have time and again proven both unwilling and unable to voluntarily change direction (24). This perspective does not downplay the significant and predictably uneven suffering that collapse will cause and is already causing. As highlighted by Andreotti and the Gesturing Towards Decolonial Futures Collective (49,50), the end of the world as we know it does not signify the absolute end, but rather the conclusion of a destructive, unsustainable and profoundly inequitable way of life (51).

Planetary health and similar risk management strategies, including resilience agendas, that aim to address the real and existential threats posed by the polycrisis without confronting the fundamental structural causes, need more than mere reform or the inclusion of new ideas and perspectives, whether indigenous or otherwise. Questioning the viability of multispecies planetary health under these circumstances is a legitimate inquiry. While my intervention is less than likley to herald an historical shift, I suggest that as long as we continue employing this concept, we must broaden our understanding of what it means to be planetary and acknowledge the social, political, cultural and geographical contingencies involved in the term.

Spivak contrasts the concept of the planetary with the idea of globalization (52). Globalization is often tied to economic interests and neoliberal capitalism. In contrast, the planetary perspective fosters an awareness of the world that is based on interrelations between beings without reducing them to mere commodities. It entails ethical responsibility and humility. Spivak invites us to recognize that we, as humans, are not the center of the universe but are instead part of a much larger ecosystem. The planetary invites us to consider our relationship to the more-than-human and the material world. This requires a radical paradigm shift from seeing the world as something humans can dominate, to seeing ourselves as part of a larger ecological system that operates on symbiosis. At the same time, the concept is tied to cultural diversity. Instead of assimilating or erasing differences, the planetary approach respects and preserves these differences while seeking meaningful exchanges across cultures. Such a shift toward a paradigm fundamentally at odds with the root structural causes of the polycrisis will inevitably destabilize these structures. Similarly, the epistemological turn toward indigenous perspectives demands nothing short of a seismic transformation – one that rejects epistemic violence, anthropocentrism, capitalism, neoliberalism, colonialism, imperialism, resilience agendas, and socio-economic inequality.

Achieving health is impossible without addressing the systemic inequalities that drive environmental degradation and climate change. This paper advocates for radical action grounded in emancipatory worlding, material(ist) justice, restitution, and multispecies wellbeing – moving beyond anthropocentric and market-based solutions to foster symbiotic, multispecies health. (53–55).

## References

[1] Braidotti R. The Posthuman. Cambridge: Polity Press; 2013.

[bibr2-17579759251378432] Haraway D. Simians, Cyborgs, and Women: the Reinvention of Nature. New York, NY: Routledge; 1991.

[bibr3-17579759251378432] Wolfe C. What Is Posthumanism? Minneapolis, MN: University of Minnesota Press; 2010.

[4] Latour B. Reassembling the Social: an Introduction to Actor-Network-Theory. Oxford: Oxford University Press; 2005.

[5] Eisenstein C . Sacred Economics: Money, Gift, and Society in the Age of Transition. Berkeley, CA: North Atlantic Books; 2011.

[bibr6-17579759251378432] Eisenstein C . Climate: a New Story. Berkeley, CA: North Atlantic Books; 2018.

[bibr7-17579759251378432] Haider LJ Rieser JPS . From reflexive co-production to diffractive co-becoming: insights from new materialism for sustainability sciences. Humanit Soc Sci Commun. 2025; 12: 787.

[bibr8-17579759251378432] Jennings B. Reconceptualizing autonomy: a relational turn in bioethics. Hastings Cent Rep. 2016; 46: 11–16. 10.1002/hast.544 26847836

[bibr9-17579759251378432] Selg P Ventsel A. The ‘relational turn’ in the social sciences. In: Selg P Ventsel A (eds) Introducing Relational Political Analysis. Palgrave Studies in Relational Sociology. Cham: Palgrave Macmillan; 2020, pp.15–40.

[10] West S Haider LJ Stålhammar S Woroniecki S. A relational turn for sustainability science? Relational thinking, leverage points and transformations. Ecosyst People. 2020; 16: 304–325.

[11] Whitmee S Haines A Beyrer C Boltz F Capon AG de Souza Dias BF , et al. Safeguarding human health in the Anthropocene epoch: report of The Rockefeller Foundation–Lancet Commission on planetary health. Lancet. 2015; 386: 1973–2028. 10.1016/S0140-6736(15)60901-1 26188744

[12] People’s Agreement of Cochabamba [Internet]. 2010 [cited 2025 July 19]. Available from: https://www.climateemergencyinstitute.com/uploads/Peoples_climate_agreement.pdf

[13] The Lancet Planetary Health. The bigger picture of planetary health. Lancet Planet Health. 2019; 3: e1. 10.1016/S2542-5196(19)30001-4 30654858

[14] Horton R Lo S. Planetary health: a new science for exceptional action. Lancet. 2015; 386: 1921–1922. 10.1016/S0140-6736(15)61038-8 26188746

[bibr15-17579759251378432] Goldberg TL Patz JA. The need for a global health ethic. Lancet. 2015; 386: e37–e39. 10.1016/S0140-6736(15)60757-7 26188749

[bibr16-17579759251378432] Smith MR Singh GM Mozaffarian D Myers SS. Effects of decreases of animal pollinators on human nutrition and global health: a modelling analysis. Lancet. 2015; 386: 1964–1972. 10.1016/S0140-6736(15)61085-6 26188748

[17] Myers SS Wessells KR Kloog I Zanobetti A Schwartz J. Effect of increased concentrations of atmospheric carbon dioxide on the global threat of zinc deficiency: a modelling study. Lancet Glob Health. 2015; 3: e639–e645. 10.1016/S2214-109X(15)00093-5 PMC4784541 26189102

[18] Martens P. Planetary health: the need for a paradigm shift. BioScience. 2023; 74: 128–129. 10.1093/biosci/biad107 PMC10977863 38560621

[19] Blau A. Defending instrumental rationality against critical theorists. Polit Res Q. 2020; 74: 1067–1080.

[20] Funk JK. Beyond instrumental rationality. For a critical theory of freedom. Estud Filosofía. 2021; 63: 91–108.

[21] Morton T. The Ecological Thought. Cambridge, MA: Harvard University Press; 2010, p.15.

[22] Ghosh A . The Nutmeg’s Curse: Parables for a Planet in Crisis. Chicago, IL: University of Chicago Press; 2021.

[23] Ghosh A. Smoke and Ashes: a Writer’s Journey Through Opium’s Hidden Histories. New York, NY: Farrar, Straus and Giroux; 2023.

[24] Zekavat M . Leveraging Satire for Environmental Advocacy: Creative Arts in the Chthulucene. Cham: Palgrave Macmillan; 2026.

[25] Ghosh A. The Great Derangement: Climate Change and the Unthinkable. Chicago, IL: University of Chicago Press; 2016.

[26] Moore JW. The Capitalocene, Part 1: on the nature and origins of our ecological crisis. J Peasant Stud. 2017; 44: 594–630.

[27] Haraway D. Anthropocene, Capitalocene, Plan-tationocene, Chthulucene: making kin. Environ Humanit. 2015; 6: 159–165.

[28] Clark H. Governance for planetary health and sustainable development. Lancet. 2015; 386: e39–e41. 10.1016/S0140-6736(15)61205-3 26188750

[29] Vallier K. Neoliberalism. In: Zalta EN Nodelman U (eds) The Stanford Encyclopedia of Philosophy [Internet]. Winter 2022 ed. Stanford, CA: Metaphysics Research Lab, Stanford University; 2022 [cited 2025 April 21]. Available from: https://plato.stanford.edu/archives/win2022/entries/neoliberalism/

[30] Watts G. Judith Rodin: on the path to resilience in planetary health. Lancet. 2015; 386: 1936. 10.1016/S0140-6736(15)61214-4 26188751

[31] Chaudhary AS . The Exhausted of the Earth: Politics in a Burning World. New York, NY: Columbia University Press; 2024.

[32] Horton R Beaglehole R Bonita R Raeburn J McKee M Wall S. From public to planetary health: a manifesto. Lancet. 2014; 383: 847. 10.1016/S0140-6736(14)60409-8 24607088

[33] Schuftan C Legge D Sanders D Nadimpally S . A manifesto for planetary health. Lancet. 2014 ;383: 1459-1460. PMID: 24766957. 10.1016/S0140-6736(14)60710-8 24766957

[34] Editorial: Planetary health for the ages. Lancet. 2022; 399: 1279. 10.1016/S0140-6736(22)00611-0 35366994

[35] Burkle FM Jr. A manifesto for planetary health. Lancet. 2014; 383: 1459. 10.1016/S0140-6736(14)60708-X 24766956

[36] Schuftan C Legge D Sanders D Nadimpally S. A manifesto for planetary health. Lancet. 2014; 383: 1459–1460. 10.1016/S0140-6736(14)60710-8 24766957

[37] Beaglehole R. Sustainable human development—but how? Lancet. 2015; 386: 1934–1935.

[38] Izdebski A Czerwiński S Jankowiak M Danielewski M Fiołna S Gromig R , et al. Unbalanced social–ecological acceleration led to state formation failure in early medieval Poland. Proc Natl Acad Sci USA. 2025; 122: e2409056122. 10.1073/pnas.2409056122 PMC12067273 40258139

[bibr39-17579759251378432] Diamond J. Collapse: How Societies Choose to Fail or Succeed. New York, NY: Viking Press; 2005.

[bibr40-17579759251378432] Redman CL. Human Impact on Ancient Environments. Tucson, AZ: University of Arizona Press; 1999.

[bibr41-17579759251378432] Ponting C. A New Green History of the World: The Environment and the Collapse of Great Civilizations. London: Penguin Books; 2007.

[bibr42-17579759251378432] Butzer KW. Collapse, environment, and society. Proc Natl Acad Sci USA. 2012; 109: 3632–3639. 10.1073/pnas.1114845109 PMC3309741 22371579

[bibr43-17579759251378432] Costanza R Graumlich LJ Steffen W (eds). Sustainability or Collapse? An Integrated History and Future of People on Earth. Cambridge, MA: MIT Press; 2007.

[bibr44-17579759251378432] McAnany PA Yoffee N (eds). Questioning Collapse: Human Resilience, Ecological Vulnerability, and the Aftermath of Empire. Cambridge: Cambridge University Press; 2010.

[45] Cronon W. Changes in the Land: Indians, Colonists, and the Ecology of New England. New York, NY: Hill and Wang; 1983.

[46] Saito K. Slow Down: The Degrowth Manifesto. New York, NY: Astra Publishing House; 2024.

[47] Ferdinand M . A Decolonial Ecology: Thinking from the Caribbean World. Cambridge: Polity Press; 2021.

[48] Latour B. Facing Gaia: Eight Lectures on the New Climatic Regime. Cambridge: Polity Press; 2017, pp.196–197.

[49] Gesturing Towards Decolonial Futures. Decolonial Futures [Internet]. ND [cited 2023 October 19]. Available from: https://decolonialfutures.net/

[50] Machado de Oliveira V. Hospicing Modernity: Facing Humanity’s Wrongs and the Implications for Social Activism. Berkeley, CA: North Atlantic Books; 2021.

[51] Quinn D. Ishmael: An Adventure of the Mind and Spirit. New York, NY: Bantam Books; 1992.

[52] Spivak GC. Death of a Discipline. New York, NY: Columbia University Press; 2003.

[53] Barca S. Forces of Reproduction. Cambridge: Cambridge University Press; 2020.

[bibr54-17579759251378432] Chakrabarty D. The Climate of History in a Planetary Age. Chicago, IL: University of Chicago Press; 2020.

[55] Haraway DJ. Staying with the Trouble: Making Kin in the Chthulucene. Durham, NC: Duke University Press; 2016.

